# Lessons for a SECURE Future: Evaluating Diversity in Crop Biotechnology Across Regulatory Regimes

**DOI:** 10.3389/fbioe.2022.886765

**Published:** 2022-05-02

**Authors:** Dalton R. George, Eli D. Hornstein, Carrie A. Clower, Allison L. Coomber, DeShae Dillard, Nassib Mugwanya, Daniela T. Pezzini, Casey Rozowski

**Affiliations:** ^1^ Department of Forestry and Environmental Resources, College of Natural Resources, North Carolina Sate University, Raleigh, NC, United States; ^2^ Genetic Engineering and Society Center, North Carolina State University, Raleigh, NC, United States; ^3^ Department of Plant and Microbial Biology, College of Agriculture and Life Sciences, North Carolina State University, Raleigh, NC, United States; ^4^ Department of Communication, College of Humanities and Social Sciences, North Carolina State University, Raleigh, NC, United States; ^5^ Department of Biological Sciences, College of Sciences, North Carolina State University, Raleigh, NC, United States; ^6^ Department of Entomology and Plant Pathology, College of Agriculture and Life Sciences, North Carolina State University, Raleigh, NC, United States; ^7^ Department of Agricultural and Human Sciences, College of Agriculture and Life Sciences, North Carolina State University, Raleigh, NC, United States; ^8^ Department of Agricultural and Resource Economics, College of Agriculture and Life Sciences, North Carolina State University, Raleigh, NC, United States

**Keywords:** crop biotechnology, SECURE rule, regulation, diversity trends, innovation, United States

## Abstract

Regulation of next-generation crops in the United States under the newly implemented “SECURE” rule promises to diversify innovation in agricultural biotechnology. Specifically, SECURE promises to expand the number of products eligible for regulatory exemption, which proponents theorize will increase the variety of traits, genes, organisms, and developers involved in developing crop biotechnology. However, few data-driven studies have looked back at the history of crop biotechnology to understand how specific regulatory pathways have affected diversity in crop biotechnology and how those patterns might change over time. In this article, we draw upon 30 years of regulatory submission data to 1) understand historical diversification trends across the landscape and history of past crop biotechnology regulatory pathways and 2) forecast how the new SECURE regulations might affect future diversification trends. Our goal is to apply an empirical approach to exploring the relationship between regulation and diversity in crop biotechnology and provide a basis for future data-driven analysis of regulatory outcomes. Based on our analysis, we suggest that diversity in crop biotechnology does not follow a single trajectory dictated by the shifts in regulation, and outcomes of SECURE might be more varied and restrictive despite the revamped exemption categories. In addition, the concept of confidential business information and its relationship to past and future biotechnology regulation is reviewed in light of our analysis.

## Introduction

Regulation of next-generation crops in the United States promises to diversify innovation in agricultural biotechnology. Similar to how CRISPR-Cas and other gene editing methods have been lauded for driving diverse innovations in crop biotech development (Ahmar et al., 2020; Gupta & Shukla, 2017; Arora & Narula, 2017; Nasti & Voytas, 2021; Gao, 2021), the new USDA SECURE rule has been framed as enhancing the capacity to bring diverse innovations to market (Hoffman, 2021; Barrangou, 2020; USDA APHIS, 2020a). Specifically, some have argued that SECURE will expand the number of products eligible for exemption (Davies and Basher, 2020; Stokstad, 2020), which will increase opportunities for resource limited developers to commercialize their products and contribute to the variety of traits being developed through biotechnology (Hoffman, 2021). However, the longstanding relationship between regulation and the diversification of crop biotechnology development is not well understood empirically. Some suggest that regulation generally has an inhibitory effect on biotechnology development *via* barriers in regulatory costs and trade limitations (Smyth, 2020; Steinwand & Ronald, 2020). Others have argued that regulation can improve biotechnology development by reducing industrial uncertainty (Hansen, 2001) and enhancing product stewardship (Mbabzi et al., 2021).

Amidst these established theoretical perspectives, few data-driven studies have taken a concrete look at regulatory submissions to understand diversity in crop biotechnology and regulation. One such study, performed by Whelan et al. (2020), provides an example of how valuable this approach can be to understanding the role of regulation in diversification trends of the types of traits, organisms, and developers present in *Argentina*’s crop biotechnology sector. Surprisingly, no such study has yet been performed in the United States. In this article, we present our own data-driven approach that draws upon data from 30 years of regulatory submissions to investigate the relationship between diversity in crop biotechnology and regulation in the United States. Our analysis produces insights on what diversity in crop biotechnology looks like under two different historic regulatory pathways, how it has changed over time, and how the implementation of new regulatory rules might impact these trends.

### Diversification and Regulation

The first objective of our study was to understand diversification trends across the landscape and history of past crop biotechnology regulatory pathways. Diversification of genetic engineering, for the purposes of our study, is defined as the breadth of organisms, genes, and traits that can be targeted in crop biotechnology development (Kumlehn et al., 2018), and the types of developers participating in commercialization. Our first step was to understand the relationship between regulatory mechanisms and proposed innovation in an empirical way. Toward this objective, we ask three questions:

1 What kinds of organisms, genes, traits, and developers have been subjected to past regulatory pathways?
2 How did the diversity within these categories change over time?
3 In what ways were the parameters of the relevant regulatory pathway(s) responsible for any diversification trend?

To answer these questions, we draw upon 3 decades of publicly available PDNS and AIR regulatory submissions. For the past 35 years of regulation, most products have been brought to market using the Coordinated Framework for the Regulation of Biotechnology (Federal Register, 2020). Under this framework, the main regulatory trigger for engineered plants was the integration of any sequences derived from plant pest organisms. This included many genes of interest for agronomically relevant traits, as well as *Agrobacterium* T-DNA sequences used as engineering tools (Hoffman, 2021). To bring a genetically engineered plant to market, developers were required to submit a “Petition for Determination of Nonregulated Status” (PDNS) through the USDA Animal and Plant Health Inspection Service (APHIS). Petitions required developers to submit extensive data to APHIS to perform a full science-based risk assessment before being granted permission to bring a product to market. Details of risk assessment outcomes were made publicly available, in addition to all relevant documents associated with PDNS submissions. However, in the late 2000s, developers began proposing and utilizing GE methods that removed plant pest sequences from the final engineered crop product (Wolt et al., 2016a). A lack of pest sequences did not trigger the requirement for a traditional science-based risk assessment, creating a gap between developer’s next-generation products and regulatory scrutiny.

In response to this gap in regulation, USDA APHIS developed the “Am I Regulated” (AIR) consultation process. Unlike PDNS, AIR was an optional process designed for developers to use when they wanted to confirm whether or not their products were exempt from regulation by the USDA. Products could theoretically be brought to market without this regulatory consultation, but developers still made substantial use of this service. AIR letters of inquiry and responses from the USDA are publicly available, providing a record of exempt products and their associated characteristics (APHIS, 2022). However, specific details on traits, genes, methods, and organisms are not always fully available in AIR letters of inquiry as companies are given the option to claim confidential business information. This is not the case in the PDNS submissions, where all data and correspondence shared between developers and regulators are a matter of public record.

### The Future as SECURE

The second objective of our study is to forecast how the new SECURE regulations might affect diversification trends in regulatory submissions and outcomes. The SECURE rule changes what types of GE crops are subject to review, which reconfigures what is eligible for regulatory exemptions. For example, plants with limited gene edits remain exempt from regulation, while those with multiple edits, multi-base-pair edits, and template-directed repair of edits are now subject to review (USDA APHIS, 2020b). Transgenic plants that recapitulate a previous combination of plant, trait, and mode of action are now exempt from regulation, regardless of whether they use methods that involve plant pest sequences, while novel transgenics are subject to review regardless of whether or not they make use of plant pest sequences (USDA APHIS, 2020b).

The review process itself has also been changed significantly as a new step called “regulatory status review” (RSR) replaces the traditional PDNS process. All submissions that undergo RSR are subjected first to an evaluation, which can take up to 180 days. This first step is designed to determine if the genetic engineering event requires a full evaluation by USDA APHIS based on “a plausible pathway to increased plant pest risk” (USDA APHIS, 2020d)**.** If APHIS determines that a plausible pathway exists, the results of the RSR would direct the developer to request that APHIS complete a full evaluation of all factors of concern related to potential increased plant pest risk. Non-regulated status is then determined from the results of this full evaluation, which APHIS estimates will take up to 15 months (USDA APHIS, 2020a).

SECURE has attracted attention from both opponents and proponents of GE crop regulation, and prognostication about how it will shape the future of agricultural biotechnology. APHIS frames the new SECURE rule as “reducing the regulatory burden for developers,” (USDA APHIS, 2020b, pg 29790) because it creates expanded categories of exemptions and reforms the review process to proceed in an expedited fashion (in some cases). Some scholars have argued that this will promote diversification (Hoffman, 2021) and democratization (Barrangou, 2020) of crop biotechnology, in part because of a greater capacity to efficiently move products through regulatory review. However, authors have also pointed to problems with SECURE self-exemption rules and non-disclosure norms. Primarily, the self-exemption rule has been critiqued as formally departing from a science-based risk assessment (Jaffe, 2020; Kuzma and Greiger, 2020), a longstanding norm in biotechnology regulation. Potential ramifications of self-exemption and non-disclosure practices under SECURE also include other issues related to international trade compliance (Grossman, 2020), domestic disclosure laws (Jaffe, 2020), and eroding public trust due to a lack of transparency in crop biotech development (Kuzma and Greiger, 2020).

It’s not clear from prior analyses how SECURE will affect the diversification of crop biotech, nor how these trends compare to what has happened under past regulatory regimes. As cited above, analyses of SECURE have applauded the revamping of safety standards to enhance commercialization (Barrangou, 2020; Hoffman, 2021) and critiqued some of the socioeconomic implications of these new rules (Kuzma and Greiger, 2020; Jaffe, 2020). However, discussions around the past and future of crop biotechnology regulation—especially around SECURE—are largely grounded in conceptual and theoretical analyses. Empirical contents of regulatory submissions are rarely, if ever, brought into analyses in a systematic fashion.

To establish a more concrete idea of how the previous exemption and review systems will compare to the new RSR landscape, we use our archival data to simulate product exemptions, expedited reviews, and full reviews under SECURE. Our goal is to analyze claims that SECURE will open up the landscape of agricultural biotechnology to a greater diversity of crops, traits, genes, and developers. In addition, there is no academic source providing an overview of what types of organisms, traits, genes, and developers have gone through regulation in the United States like there is for other countries, such as *Argentina* (Whelan et al., 2020). We suggest that now, at the point of transition to a new regime, is the ideal time to develop such a data resource for the United States. Doing so will allow the research community to perform more grounded analyses of past regulatory regimes and understand how future innovations are likely to fare under SECURE.

## Materials and Methods

Our data collection was driven by a content analysis on documents from two public sources: USDA APHIS petitions for determination of nonregulated status (PDNS) and “Am I Regulated?” inquiry letters (AIRs). Both PDNSs and AIRs are public documents made available *via* PDF copies of the original submissions and agency responses on the website of the USDA Biotechnology Regulatory Service. Information was manually extracted by reading through each submission and coded into an Excel spreadsheet according to a coding scheme devised by the researchers to capture various kinds of data from the archival documents (described below). Each document was read and initially coded by one investigator, with coding decisions reviewed by a second investigator for reasons of inter-coder reliability and accuracy. Outside literature and related regulatory filings were consulted for clarity when needed. Target organisms, engineering methods, traits conferred, and genes affected were collected for all entries where available. The nature of all entities submitting petitions was also reviewed and categorized after data collection was complete. Where data of a certain type was submitted for review but not publicly available, a value of “redacted” was recorded (see Supplementary Appendix S1).

Target organism data of two types was collected. First, the organism identity at the species level was recorded. Second, a crop category based on the USDA Agricultural Census categories was determined based on explicit inclusion in the category or similarity to existing members. We added categories for organisms not present in the USDA system: engineered bacteria and fungi were grouped as “microorganism,” and timber crops were categorized under “forestry.” An “Other” category contains submissions such as the five separate glowing plants sent to AIR. The organism categories used were (in alphabetical order): fiber, forage, forestry, fruit, grain, microorganism, nursery and flowers, oilseeds and other commodities, other, and vegetable.

Engineering method data of three types was collected. First, the genetic transformation method used to introduce transgenes (including editing constructs) was recorded. Transformation methods included *Agrobacterium*, biolistic, and several less common methods. Null sergeants of engineered organisms were also recorded. Second, the nature of the genetic engineering process with regard to the gene of interest’s function in producing a trait was encoded as overexpression, silencing (including sense and antisense suppression and RNAi), and gene editing. Finally, where gene editing was used, the type of editing technology was recorded.

Trait data of two types was collected. First, “Trait” consisted of a ∼1–3 word description of the intended trait as given in the regulatory submission. The description includes the targeted trait and the direction of change from the wildtype, e.g., “reduced toxicity.” In some cases, proximate and distal traits were reported, and these were encoded together separated by a “_,” e.g., “altered ethylene synthesis_early flowering.” Traits were recorded in separate columns and individual values of yes or no were recorded for each trait to allow for the presence of multiple traits in a single plant. The functional category of each trait was encoded as: agronomic; herbicide resistance; resistance to bacteria, fungi, viruses, or insects; product quality; or other.

Gene data of two types was also collected. First, each gene engineered in each submission was recorded. Second, the species of origin of the engineered gene was determined and recorded alongside the gene itself. To record genes engineered with different homologs or versions across different regulatory submissions, a column containing a generic acronym of the gene name was created, and the distinctive gene name and species of origin as given in each submission was entered as a datapoint in that column. For example, editing of specific polyphenol oxidases in potato (*Solanum tuberosum*) and apple (*Malus domestica*) was recorded as values of “St-Ppo5” and “Md-Ppo2” under the single column “PPO-1.”

Developer type was determined as government, university, non-governmental organization, small to medium enterprise (SME), or large commercial enterprise (LCE). LCEs are defined as companies who employ 250 or more individuals, and SMEs are those companies who employ less than 250 individuals (OECD, 2021). When necessary, additional literature, regulatory filings, and media reports were consulted to differentiate between the two types of commercial entities.

In our analysis of the categories for species, developer, and trait, we determined the percentage of submissions falling under each crop category, entity type, and trait functional category, respectively. We also determined the average number of traits per submission and genes per trait by dividing the sum of all traits by the number of submissions and the sum of all genes by the sum of all traits, respectively, for each year in each process and plotted these against time. To enable direct comparison of submissions to PDNS and AIR, we controlled for differences in the timespan and total number of submissions between the two processes. First, we limited submissions to both processes to only those made from 2011 to 2020, the time period in which both AIR and PDNS were active. Then, we removed submissions where information in a given category was not disclosed due to a claim of confidential business information. Then, we normalized our diversity categories (total unique developers, organisms, traits, and genes) to the total number of submissions to each pathway in this time period. This gives an indication of how diverse submissions are to each pathway in terms of how often a new submission involves a data point that has not been seen before, independent of the total volume of submissions. These simple “diversity ratios” may be interpreted as: “on average, *n* unique data points are added to category X with every new submission.” In addition to controlling for differences in submission rate and timeframe, the goal of using these ratios is to have a metric that reflects the relationship of the regulatory processes themselves to the broader biotech development system that generates submissions.

An important part of this study was to use the past regulatory submission data to forecast how the biotech regulatory landscape under the new SECURE rule would treat different product types. To do this, we analyzed how many of the 301 regulatory submissions from AIR and PDNS would have qualified for an exemption category or proceeded to RSR had SECURE been in place at the time of their development. For analysis of projected outcomes under the SECURE rule, we characterized each entry in our database as a predicted exemption when corresponding to a set of values, as specified in Table 1 (see Methods Supplement). Outputs indicated which events in AIR and PDNS submissions would theoretically have been “regulated” and required an RSR or “exempted” based on a number of categorizations for novel products. Categorizations that would indicate an exempt product include: 1) if a product contains a single, non-template-guided gene edit, 2) a product is a null segregant of an engineered line with no remaining engineered genes, 3) a product contains an insertion of a single gene from the same species as the host organism or 4) a product is a repetition of a previously deregulated plant-trait-mode of action combination.

**TABLE 1:** Definitions of terms used in this article.

Term	Abbreviation	Definition
Am I Regulated? inquiry letters	AIR	A regulatory pathway where genetic engineering developers could submit a letter for review by APHIS to determine if their product was a regulated article. Discontinued in June 2020 and later replaced by SECURE.
Confidential business information		Competitive information pertaining to trade secrets, intellectual property, or other protected assets which are often redacted from AIR letters.
Diversity in biotechnology		The breadth of organisms, genes, and traits that can be targeted in genetic engineering development.
Genetically engineered	GE	The usage of biotechnology to alter or otherwise manipulate the genetic makeup of an organism.
Large Corporate Enterprise	LCE	A company which employs more than 250 individuals.
Petition for the Determination of Nonregulated Status	PDNS	A regulatory pathway where genetic engineering developers could submit a petition to APHIS to determine if a plant engineered with a plant pest posed a plant pest risk. Now replaced by SECURE.
Regulatory Status Review	RSR	A regulatory pathway where genetic engineering developers can request a review of a new genetically engineered plant which has not previously been given nonregulated status.
Small-to-Medium Enterprise	SME	A company which employs less than 250 individuals.
Sustainable, Ecological, Consistent, Uniform, Responsible, and Efficient ruling	SECURE	Revisions to APHIS’s biotechnology regulations which reduce barriers to genetic engineering products which do not pose a plant pest risk. Became fully effective in Fall 2021.

## Results

### Overview of AIR and PDNS

**FIGURE 1:**
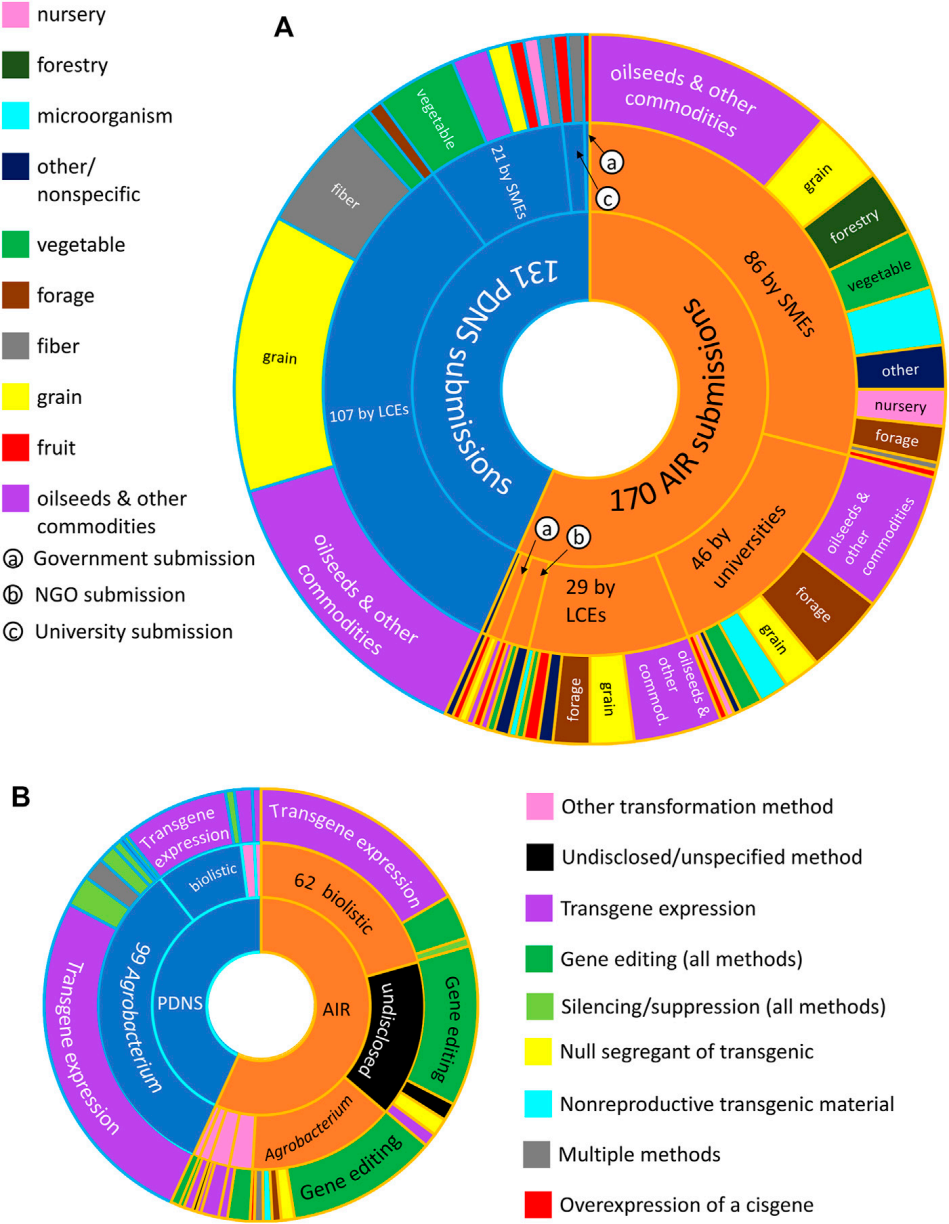
Overview of different facts of diversity across AIR and PDNS. **(A)** Total submissions (innermost ring) and number of submissions contributed from each category of developer (center ring). The outermost ring shows the number of submissions in each crop category submitted by each category of developer. **(B)** Total submissions (innermost ring), transformation methods (center ring), and engineering methods (outermost ring) used in submissions to each regulatory pathway. Submissions containing undisclosed/redacted transformation methods (center ring) or engineering methods (outer ring) are shown in black.

When comparing an overview of all 301 submissions across the full time periods in which each process was active, it is notable that AIR attracted more total submissions (170) than PDNS(131) despite being active for a much shorter time. The AIR process appears more diverse in all aspects of diversity that we considered. In terms of developers (Figure 1A), AIR received submissions from 66 separate entities spread across all five developer categories. SMEs and universities were the largest contributors of AIR submissions with 86 and 46, respectively, followed by LCEs with 29 submissions. PDNS received submissions from 38 unique entities, dominated by 107 submissions from LCEs, followed by 21 from SMEs and a combined five submissions from government entities and universities.

The distribution of submissions across crop categories also differs with regulatory process and developer type (Figure 1A). Submissions by LCEs in the top two crop categories (“grains” and “oilseeds and other commodities”) account for more than half of all submissions to PDNS. In contrast, AIR submissions are not dominated by submissions of any one crop type; no crop category makes up more than 50% of all submissions or more than 50% of the submissions from any developer category. To reach the simplest possible majority of AIR submissions, it is necessary to combine submissions across four crop categories and three developer categories.

Engineering methods also varied across AIR and PDNS (Figure 1B). As expected, the great majority of PDNS submissions involved expression of one or more transgenes. Gene editing was the most common genetic engineering event in AIR submissions, followed by transgene expression. In terms of the transformation method, the great majority of submissions to PDNS used *Agrobacterium*. No one transformation method accounted for a majority of AIR submissions, with a slim plurality using biolistic gene delivery. A significant portion of AIR submissions did not disclose their transformation method or were not tied to a specific method; “undisclosed/unspecified” was the second most common classification after biolistic transformation. Notably, the transformation method varied significantly with the type of genetic engineering event: the great majority of gene-edited AIR submissions used *Agrobacterium* delivery, while the great majority of transgene-expression submissions used biolistics.

**FIGURE 2:**
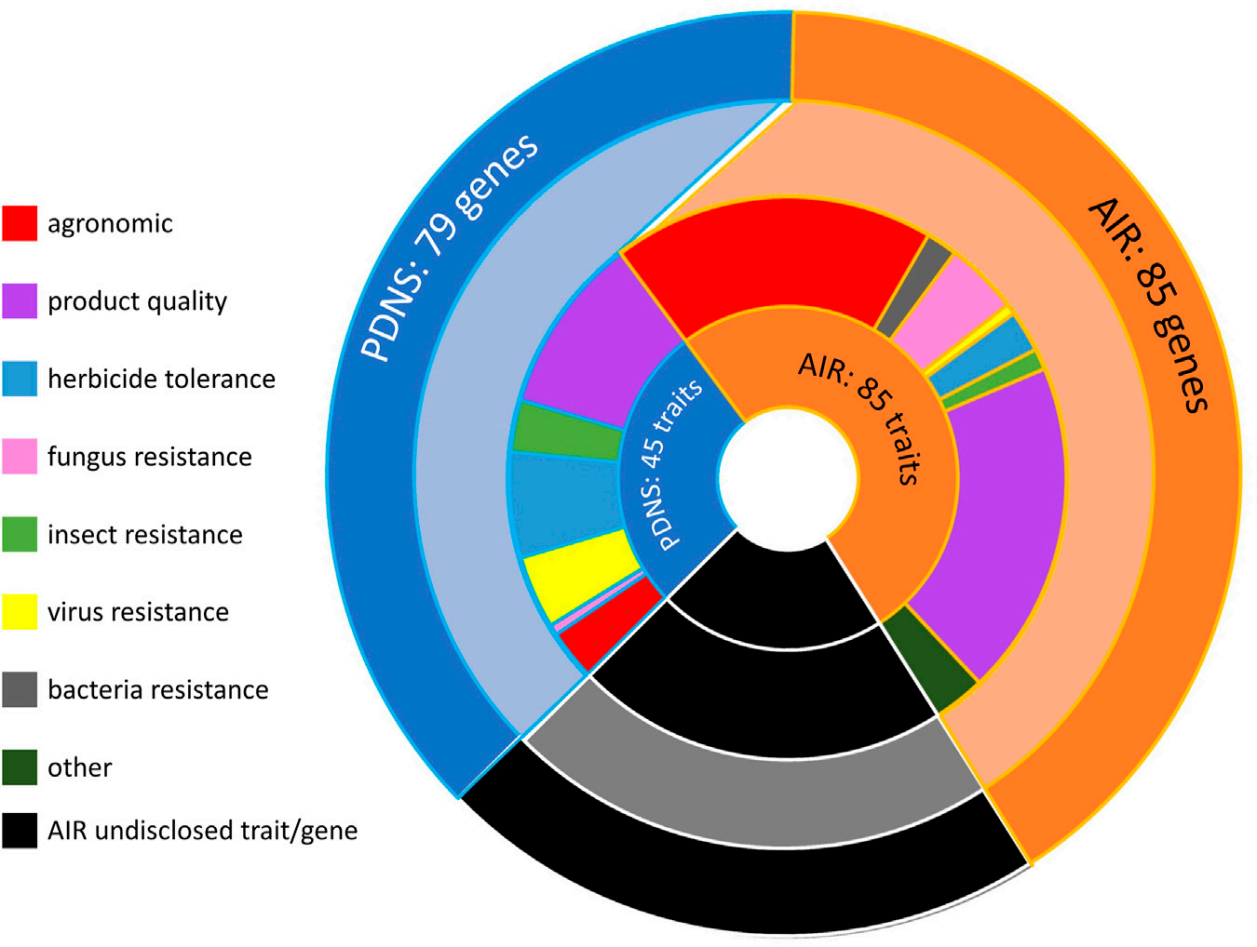
The distribution of traits, trait categories, and total genes across all AIR and PDNS submissions. The innermost ring shows the number of unique traits engineered in submissions to each pathway. The next-innermost ring shows the number of unique traits engineered in each trait category across the two pathways, and the outermost ring shows the total number of unique genes engineered across all submissions to each pathway. AIR submissions in which the gene or trait of interest was redacted are represented in black. By their nature it is not possible to know the exact number of redacted genes or traits; this section of the graph is drawn proportionally to the number of submissions containing only redacted trait information.

A greater number of total unique traits and genes was submitted to AIR than to PDNS (Figure 2). The difference in trait numbers was 85 total for the AIR submissions, and 45 for the PDNS, while for genes it was 85 for AIR and 79 for PDNS. *Bt* transgenics submitted to PDNS are a notable contributor to the fact that the number of genes is similar across regulatory systems despite the difference in the number of traits. Sixteen discrete *Bt* genes were recorded in our dataset but contributed to only five discretely categorized insect resistance traits. As shown in Figure 1B and Figure 2, the genes and/or traits in a significant portion of AIR submissions are redacted. Because redacted submissions may target a gene or trait that is the same as an existing submission, or may target multiple genes and traits, it is impossible to know the exact values underlying these categories.

### Comparing Diversity in AIR and PDNS

While a useful overview, the utility of comparing the two regulatory processes using raw data like in Figures 1, 2 is limited by: AIR attracting a greater total number of submissions than PDNS, and PDNS (1991–2021) existing for nearly three times as long as AIR (2010–2021). Differences in the available technology at different times and the fact that AIR was not available as an alternative for most of the lifespan of PDNS could impact comparisons done with the raw data.

**FIGURE 3:**
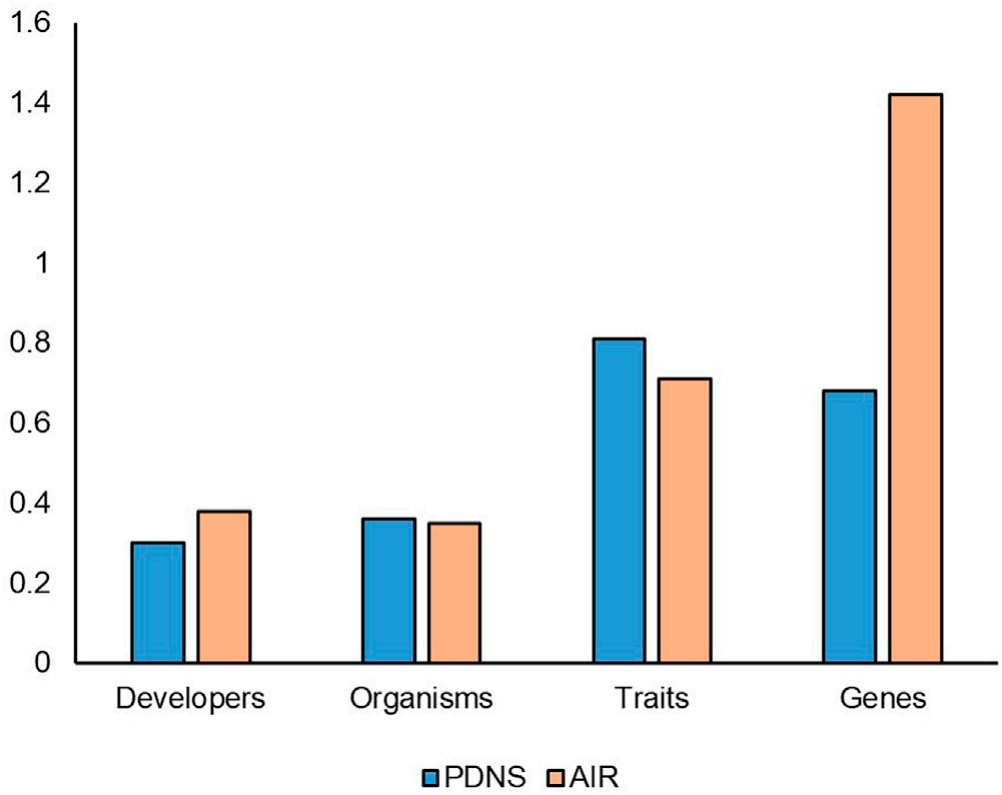
Diversity ratios from 2011 to 2020. The number of submissions accounted for in this timeframe include all of the AIR submissions, but only the last 10 years of PDNS submissions. This timeframe reflects the years where the AIR & PDNS processes existed simultaneously. Redacted submissions in AIR are omitted from the ratio calculation.

The diversity ratios for submissions from 2011 to 2020 (Figure 3) give a different view of the comparison. In addition to controlling for time, these values also control for the confounding factors mentioned above by normalizing to the total number of submissions (minus AIR submissions redacted for that category). As shown in Figure 3, in a direct comparison AIR no longer broadly leads PDNS. For Genes, the AIR pathway is still more diverse than PDNS. Notably, Genes have a diversity ratio greater than one in AIR, indicating the fact that on average more than one new gene was engineered for each new submission. The Developer diversity ratio is also higher for AIR (0.38) than for PDNS (0.3) showing that a new submission to AIR is more likely to come from a first-time developer. In Organisms, PDNS and AIR submissions are equal. PDNS submissions contained 0.36 new species per submission, while for AIR this was 0.35 species per submission. Notably, PDNS now clearly exceeds AIR in trait diversity, with developers submitting 0.81 new traits for every submission, while AIR submissions included 0.71 new traits each. Taken together, the results show that, after accounting for timespan and submission volume, the comparison of diversity in regulatory systems does indeed vary considerably across different facets of biotechnology. In some of these facets PDNS exceeds or equals AIR in its relationship to diversity.

The results in Figure 3 also indicate that for several categories, the PDNS pathway must have had a greater level of diversity in the latter part of its existence from 2011 to 2020 than in the earlier years excluded from the time-corrected data in Figure 3. To further examine change over time in the PDNS process, we compared the diversity ratios for all 30 years of PDNS submissions split into three 10-year periods.

**FIGURE 4:**
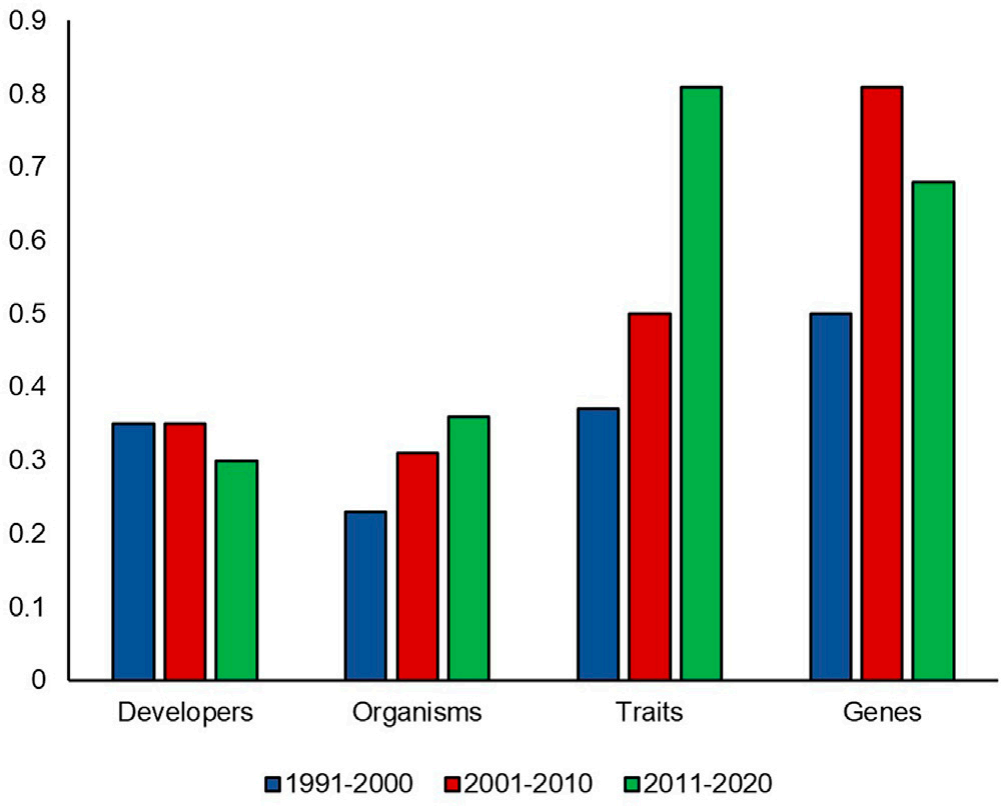
PDNS diversity over time. Diversity ratios in each category were calculated for each 10 year timespan of the PDNS across all categories of interest.

Figure 4 demonstrates how diversity in submissions to PDNS has changed over time. For organisms and traits, diversity ratios have steadily increased over time from 1991–2020. For organisms, the ratios increase from 0.23, 0.31, to 0.36. For traits, the ratios increase from 0.37, to 0.5, to 0.81. Developer diversity remains relatively consistent at 0.35, 0.35, and 0.30, while gene ratios start at 0.5, increase to 0.81, but then drop to 0.68. The PDNS diversity over time shows that the timeframe of submissions–potentially reflecting differences in agricultural needs and/or the state of technology–alters the makeup of submissions, varies in its effect across diversity facets, and validates the need to control for it when comparing PDNS to AIR.

### Traits, Crops, and Developers in Detail

To better understand what underlies the differences in different facets of diversity across submissions, we analyzed more detailed data within each category. Here, we show summary statistics and time-controlled comparisons of PDNS and AIR.

**FIGURE 5:**
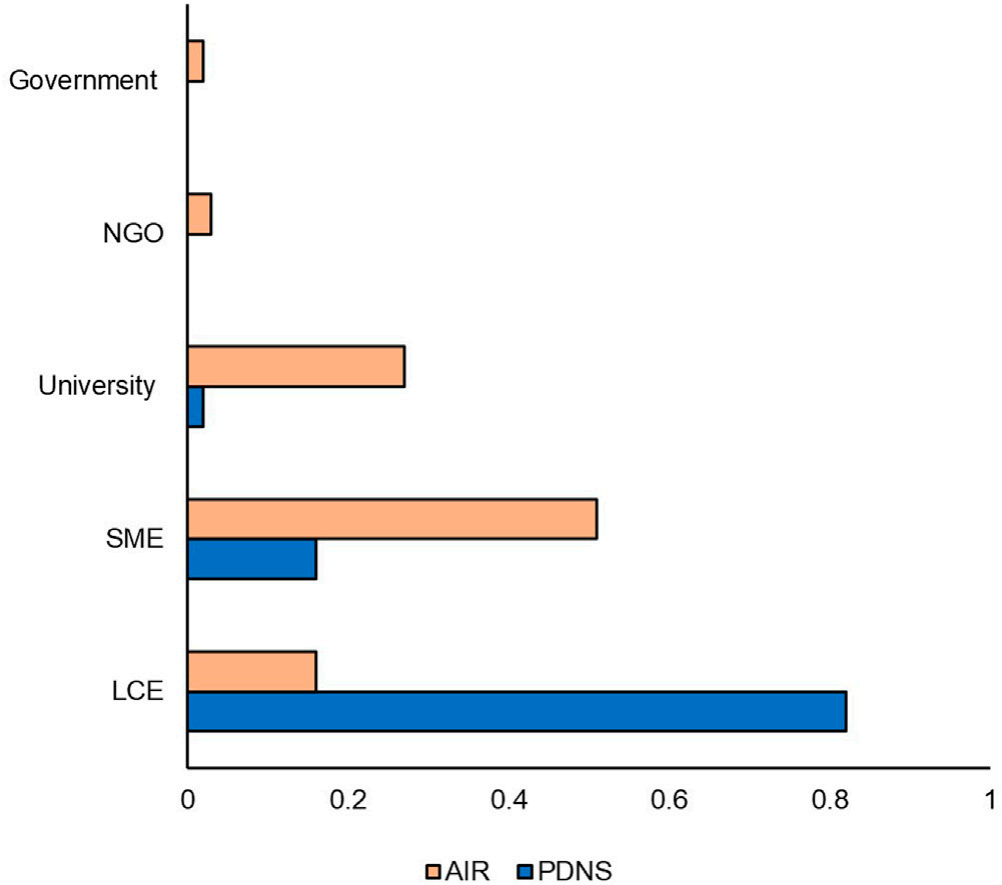
Developer comparison. Percentages indicate the share of the total number of times that category occurred in the data set. Comparison is from 2011 to 2020.

While Figure 3 showed that over 2011–2020 AIR was slightly more diverse in total developers per submission than PDNS, Figure 5 shows differences in the makeup of participants contributing to those figures. Figure 5 shows the percentage of submissions to AIR and PDNS from 2011 to 2020 contributed by each type of developer. Participants in AIR belonged to more categories, were more evenly distributed, and were dominated by different developer types than PDNS. 82% of submissions to PDNS were from LCEs. In contrast, the majority of submissions to AIR came from SMEs but this category did not as strongly dominate the field, contributing 50% of submissions. The share of submissions to AIR from universities was also more than ten-fold greater than in PDNS, making universities the second-most prevalent contributor of AIR submissions.

**FIGURE 6:**
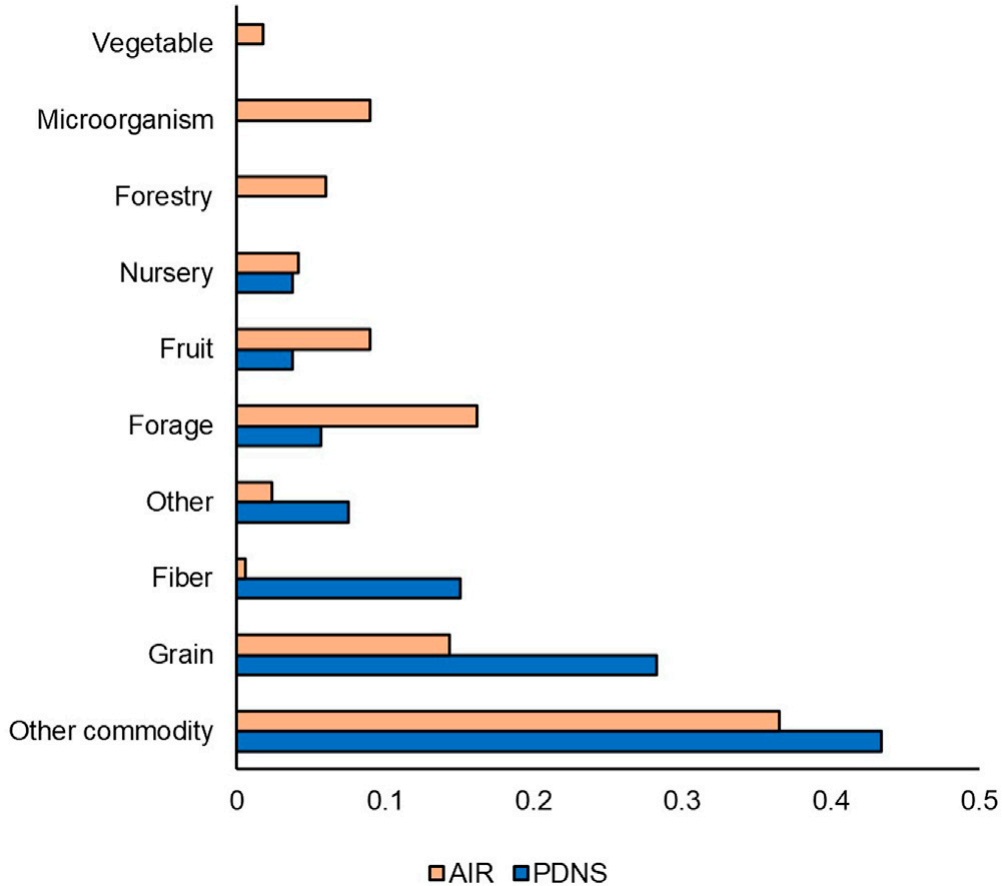
Organism comparison. Unique organisms were categorized based on USDA Quick Stats & ERS crop designations, and researcher-assigned categories to cover organisms not addressed by USDA designations (i.e. microorganisms). Percentages indicate the share of the total number of times that category occurred in the data set. Comparison is from 2011 to 2020.

Across all PDNS and AIR letters, our study registered a total of 81 unique organisms, with 62 coming from the AIR letters and 19 from the petitions. Only one species, chicory, appeared in PDNS but not AIR. In Figure 6, we compared the percentage of submissions to each regulatory process contributed by each of the crop categories from 2011 to 2020. AIR submissions were more diverse than PDNS, containing at least one submission to each of the ten categories. AIR submissions were also more evenly distributed: 87% of PDNS submissions but only 52% of AIRs came from the categories of ‘Grain’ ‘Other Commodity,’ and ‘Fiber’ which contain traditional row crops. A greater fraction of AIR submissions were devoted to the categories of Microorganism, Nursery, Fruit, Forestry, Forage, Other Commodity, and Other than in PDNS submissions.

**FIGURE 7:**
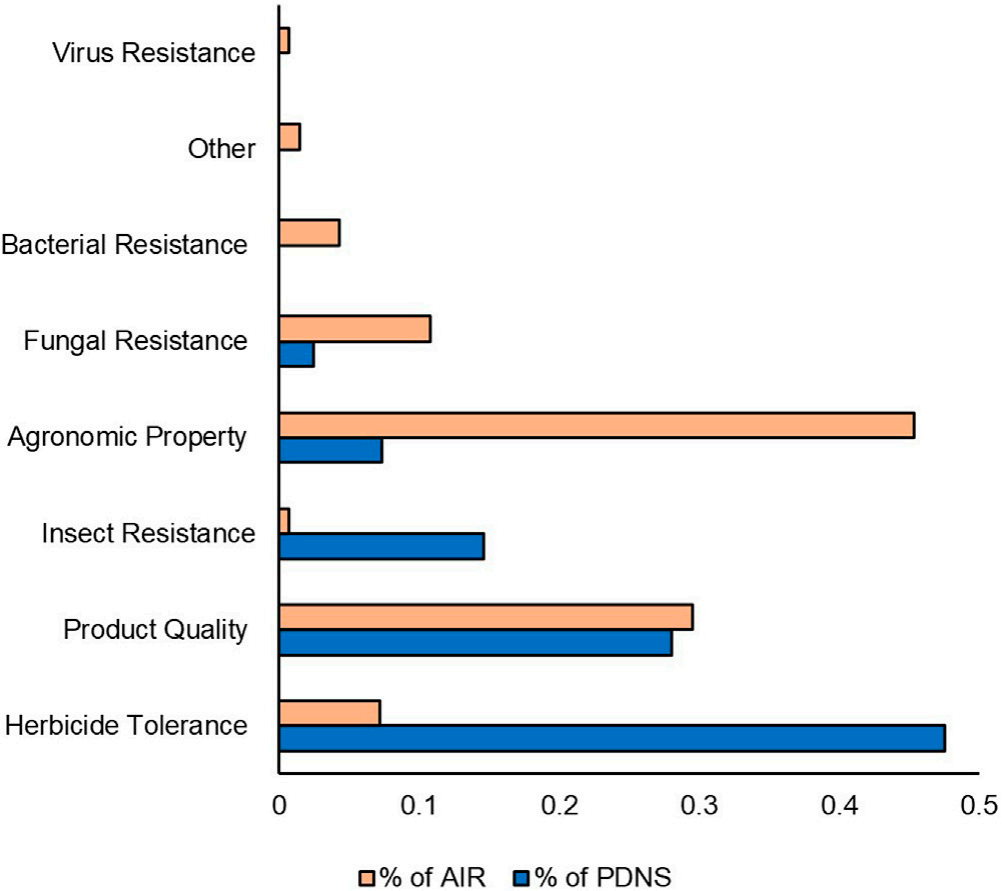
Traits comparison. Traits were categorized using common USDA ERS designations. Percentages indicate the share of the total number of times that category occurred in the data set. Comparison is from 2011 to 2020.

Next, we compared the time-controlled dataset for differences in trait categories (Figure 7). Figure 7 shows the percentage of submissions containing each trait category to AIR and PDNS from 2011 to 2020. Herbicide tolerance and insecticidal traits are much more prevalent in the PDNS data than AIR, making up 42.9% and 21.5%, as opposed to 7.2% and 1.4% respectively. In the AIR letters, agronomic properties were the most prevalent trait category, making up over 35% of the total reported trait targets. The trait category where both sets of articles were most similar was in product quality, which made up 29.5% of the AIR articles and 21.5% of the PDNS.

Lastly, our results determined how many distinct genes were engineered across both datasets. In total, 79 individual and unique genes were engineered in PDNSs and 85 genes were engineered in AIRs. We noted that the total unique genes number was significantly lower than the total submissions in both cases for different reasons. First, many PDNS submissions are focused on the same gene. Among PDNS the herbicide resistance genes *EPSPS* (glyphosate) and *Bar* (glufosinate) were the most commonly used, being inserted in 24 and 31 events respectively. We also noted that we treated 16 separate *Bt* toxins as individual genes in accord with their structure-function relationship and the approach taken by USDA in establishing Plant-Trait-MOA categories (USDA 2021). Second, in the AIRs, many submissions redacted the specific gene targeted. Among AIRs, 110 submissions did not disclose the identity of one or more genes. However, given the strong diversity ratio for the AIR gene category when using only submissions with identified genes (Figure 3), we expect that there are many more unique genes present in the products submitted to AIR.

### Forecasting Future Regulations

**FIGURE 8:**
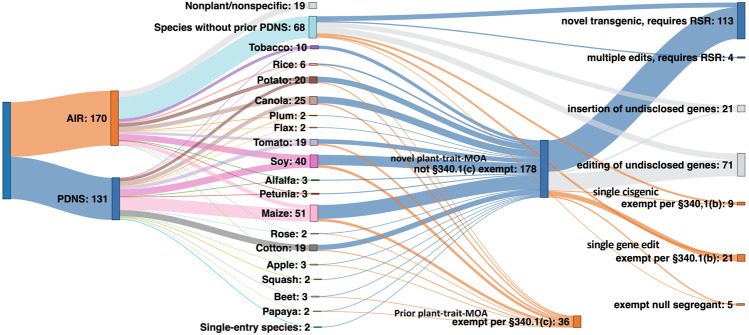
The theoretical regulatory pathway of 30 years of US agricultural biotechnology submissions had they been considered under SECURE at the time of development. Starting from left: submissions under AIR and PDNS, the individual species they contain, and their projected exempt or regulated status under SECURE.

An important part of this study was to use the past regulatory submission data to forecast how the biotech regulatory landscape under the new SECURE rule would treat different product types. To do this, we analyzed how many of the 301 regulatory submissions from AIR and PDNS would have qualified for an exemption category or proceeded to RSR (see Supplementary Appendix S2). Figure 8 shows the projected outcomes of how submissions to AIR and PDNS might have been evaluated under SECURE.

Nineteen AIR submissions pertained to general technologies or to non-plant organisms, and were therefore excluded from the total analyzed submissions. Of the remaining 283 combined submissions, 41% (117 events) would clearly have proceeded to regulatory status review. 71 events, or 25% of the total, would have been exempt from review. The 340.1(c) exemption for new events of previous plant-trait and mode-of-action combinations submitted through the PDNS process contributed the majority of exemptions, with 340.1(b) exemptions for single gene editing events in AIR letters the next most prevalent.

The remaining 34% of all submissions contained too little information to establish with clarity whether an exemption would apply under SECURE, or if the submission would have proceeded to RSR. All of these submissions originated from AIR letters, and ambiguity was due to the level of redaction due to claimed confidential business information. Excluding all redacted AIR submissions reduces the total number of submissions in the analysis to 190. Out of this total, 59% (113) would be projected to require RSR under SECURE, while 41% (78) would have qualified for an exemption.

Interestingly, these results suggest a slightly higher number of crop biotechnological events would have been subject to at least the first stage of RSR than the PDNS pathway had SECURE been in place from 1991 to the present day. It is important to note that this result might underestimate the proportion of exemptions because it includes all PDNSs while excluding 2/3 of AIRs, which show a much higher ratio of exemptions. If, rather than being excluded, the redacted gene-edited and cisgenic AIRs are instead estimated to be exempt at the same rate as unredacted submissions in their respective categories, an additional 68 exempt and 24 nonexempt events are added. This reduces the total number of submissions projected to require RSR to 48% (137), and increases exemptions to 52% (146).

## Discussion

Our study conducted a data-driven examination of anecdotal observations on the regulation of crop biotech. Our aim was to evaluate claims about the relationship between regulation and diversity in crop biotech, and provide more carefully defined metrics that can be used in future work. Our results show that diversity in crop biotech does not follow a single trajectory dictated by shifts in regulation.

AIR and PDNS are very different pathways in terms of process, and at first glance it might appear that AIR submissions would be more diverse than the PDNS. Yet when we defined diversity as the breadth of unique traits, organisms, developers, and genes passing through a regulatory pathway per submission, and compared AIR and PDNS over the period of the past 10 years where both were active, we found only slight differences in overall diversity (Figure 3) despite a large difference in the volume of submissions (Figure 1). Although AIR submissions contained more total members of each diversity category, most of this effect is explained by the fact that AIR simply attracted more submissions than PDNS. Combined, these results suggest that while AIR allowed inventions to more rapidly accumulate in the market, submissions directed to this regulatory pathway were not drastically more or less likely than in PDNS to involve a truly unique developer, organism, or trait.

AIR had a greater ratio of genes to submissions than PDNS. This is notable given that the same pattern is not seen in the ratio of traits to submissions (Figure 3), where PDNS slightly exceeds AIR. This suggests that AIR submissions engineered different genes to achieve the same trait, or are more likely to engineer multiple genes to achieve a trait. One obvious source of this effect is the inclusion of gene editing in only AIR data and its relationship to species. Each instance of editing the homolog of a gene in a new species was recorded as an additional engineered gene, while instances of inserting a previously used transgene into a new species were not considered to involve an additional engineered gene. It may also be the case that use of gene editing to achieve a trait genuinely requires targeting of a more diverse set of genes, or of additional genes, compared to transgenic methods. A more rigorous investigation of the genetic technology underlying these submissions, potentially accounting for incremental alterations to transgenes of interest over time and for the effect of regulatory and other noncoding sequences, may resolve the patterns that our preliminary results suggest are present.

We also observed that for organisms and traits, the PDNS submissions demonstrated a steady increase in diversity ratios over time (Figure 4). This is consistent with the fact that PDNS is more comparable to AIR in the timeframe of 2011–2020 (Figure 3) than over the full timeframe (Figures 1, 2). This also suggests that, regardless of regulatory pathway, diversity in organisms and traits targeted by agricultural biotechnology and submitted to regulation was increasing. We hypothesize this is reflecting a continuous increase in expertise and application over time, which are not held static by the regulations in place at a given time. This is potentially a key point in gauging the impact of regulatory barriers on the overall progress of the field. Many contributors to advancement in biotechnology are academic and non-US based researchers whose work is not directly affected by US regulations that govern commercialization.

In contrast to the relative similarity of AIR and PDNS in the most broad measures of diversity, important nuances did appear in the specific types of developers, traits, organisms, and genes present across the different pathways. For developers, it was clear from our results that LCEs were much more likely to use the PDNS pathway, while SMEs and universities were much more likely to use AIRs. For traits, PDNS submissions showed a greater incidence of products aimed at insect resistance and herbicide tolerance, while the AIR letters were much more focused on agronomic properties (Figure 7). Lastly, for organisms, both PDNS and AIR were comparable for commodities, but varied across other crop categories like grain, forage, vegetable, and fiber crops (Figure 6).

These differences reflect an interesting feature of the interplay between the parallel biotechnology regulatory pathways that have been in place for the last decade. The LCEs that dominate transgenic submissions to PDNS did not abandon this work in favor of gene editing, nor did they assume a parallel dominant position in AIR submissions commensurate with their share of PDNS. This suggests that the draw of a lower regulatory barrier in AIR acts differently on different types of developers and, as has been previously suggested **(** Hoffman, 2021), does attract a significantly greater share of smaller developers who are not invested in the legacy traits of herbicide tolerance and insect resistance. Even though submissions to AIR are only slightly more likely to come from a new developer, they are much more likely to come from an SME or university.

To better understand how SECURE will alter regulation of biotechnology, we subjected the combined body of past PDNS and AIR submissions to simulated regulation. We determined their eligibility for various exemptions or requirements had they been submitted for review under SECURE, based on the technological characteristics we recorded. We found that while a significant number of submissions changed from exempt to regulated and vice versa, the end result was not a drastic shift in either direction. The real-world data included 131 products deregulated *via* PDNS, 165 confirmed exempt from regulation *via* AIR, and five found to be regulated products *via* AIR consultation. Our simulated regulation of these products under SECURE led to 113 regulated products, 71 exempt products, and 92 products with status we could not determine due to redacted technical information in the AIR letters. Even if all of the 92 uncategorized products, which mostly resulted from gene editing, are assumed to be exempt, this provides a final ratio of 40.9% regulated to 59.1% unregulated products in our theoretical SECURE regulation, as compared to 43.2% regulated and 56.8% unregulated products in the set of biotechnology products regulated through PDNS and AIR. Thus, while much of the discussion on SECURE has related to its regulatory exemptions, our results show that its impact when applied to the actual agricultural biotechnologies regulated in the US to date ranges from roughly equivalent to more restrictive than the prior PDNS/AIR parallel system in regard to exemptions.

### Future Work on SECURE

Our investigation demonstrates that insights into the relationship between diversity and regulation can be garnered from studying official submissions to regulatory pathways, and that having transparent data is key to performing high quality analysis. Therein lies significant challenges for future work that might study the relationship between SECURE and crop biotechnology diversity. A surprising finding in the AIR letters was the high prevalence of confidential business information claims to avoid disclosing genes, methods, and traits, sometimes all in the same submission. At least one redaction from one of these categories was found in over half of AIR letters. We suggest that this indicates an overarching interest in privacy on the part of biotechnology developers.

The ability to withhold information that impacts competition and intellectual property is a potentially overlooked factor in discussions about what attracts developers to a regulatory pathway, and may be overshadowed by discussions on regulatory review processes and the barriers they empose. Discussion on the biotechnology regulation-innovation relationship has focused on the burden in terms of costs, time, and data. Looking forward, we should consider the indirect effect of enforcing disclosures on regulatory choices. Protecting competitive information may be as or more important than other costs when biotechnology developers choose to orient their technology towards a particular regulatory path.

An interesting aspect of confidential business information redaction in past regulation is that it was, in theory, permitted in PDNSs under the same set of justifications used for AIRs which specifies “genotypes, phenotypes, donor organisms, gene names, gene description, and transformation method” as prospective confidential business information (USDA APHIS, 2020c7 CFR 340.6). The greater degree of disclosure in PDNSs may be a product of administrative decision making on the part of USDA, strategic choices by developers, or simply a tendency to follow the example of earlier submissions. SECURE’s RSR also operates under these general rules, and an essential component of the future under SECURE will be whether RSR disclosures tend to treat confidential business information more similarly to AIR letters or PDNSs. If RSRs permit greater confidentiality than PDNSs, this will provide a means of comparing the relative importance of regulatory burden and confidentiality in shaping biotech developers’ incorporation of regulatory effects into their technology development decisions.

Regardless, it is unlikely that SECURE will actively increase the extent of disclosures made by biotech developers relative to past regimes, and it certainly does not mandate this practice. We may therefore experience a hidden diversity in crop biotech development, where many different novel products enter the market, but without the public being notified in any meaningful way (Kuzma and Greiger, 2020). This will make future evaluation of agricultural biotechnology difficult, and may degrade public trust due to a lack of transparency and engagement (Kuzma, 2018). The use of exemptions and truncated reviews also raises questions concerning accountability, and what happens in the case of mistakes and unintended consequences. All stages of SECURE are oriented towards evaluating the trait and engineering method of interest as they are described to the USDA. However, skipping extensive regulatory review also reduces the opportunities for crop developers to confirm that the genetic makeup of their product is exactly as intended.

For developers taking advantage of exemptions and first-stage RSR, this is potentially a high-stakes proposition. An “invalid determination,” e.g. misidentifying a known genetic change as exempt or releasing a product with additional unintended genetic changes that alter its regulatory category, can result in enforcement actions by APHIS including fines up to $1,000,000 and seizure of materials, as well as potential liability (USDA APHIS, 2020c 7 CFR 340.6, USDA APHIS, 2020b). These instances are not uncommon in standard plant engineering methods, and detection of off-target editing and silent gene insertions is frequently more difficult than engineering the trait of interest (GelvinGelvin 2003; Wolt et al., 2016b;^2017^; Zhang et al., 2018). How enforcement will work in practice is yet to be seen, especially in cases where some form of regulatory review for events later found to be misidentified has been undertaken by the government. However, pursuit of regulatory reassurance by choosing to submit to more extensive regulation than the minimum required can slow developers’ path to commercialization and potentially impact their competitiveness. This may be especially important for smaller developers that have fewer resources and that, according to our data (Figures 1, 5), are more likely to use exempt engineering methods.

We suggest that there is a combined solution to the problems of encouraging transparency, avoiding unintended engineering events, and enabling a diversity of engineering applications to be pursued by smaller developers and nonprofits. Kuzma and Grieger (2020) writing on this topic proposed a novel, voluntary, non-governmental system in which developers are incentivized to disclose basic details about their products in exchange for a certification that would “signify that the biotech crop producer is striving to become more transparent and trustworthy according to community-derived standards” (pg 917). This system, termed “CLEAR-GOV”, would exist as a nonprofit staffed by experts in the field, and use the information contributed by developers in exchange for certification to construct a database for future academic work, public availability, and engagement (Kuzma and Greiger, 2020). We support this approach, and note that disclosure is not only a matter of transparency and building public trust, but could also be very beneficial to biotechnology developers’ practical ability to operate under SECURE.

Our results suggest that a strong interest in privacy on the part of biotech developers leads them to often opt against transparency when given the choice in regulation. Participation in a voluntary transparency-focused initiative may therefore require an inducement that goes beyond a certification. The same body of developers opting for secrecy in past AIRs is also skewed towards less experienced and smaller entities. These developers benefit materially from understanding their own new technology’s regulatory position and real-world utility, which will be much easier if they have access in uniform fashion to technical parameters of decisions and review undertaken by USDA that go beyond the minimum required by law. A transparent reporting system such as CLEAR-GOV could therefore be built on the additional strength of providing information that is valuable to developers themselves in pursuit of novel innovations, while simultaneously serving the ends of good governance.

## Data Availability

Publicly available datasets were analyzed in this study. This data can be found here: Petitions for Determination of non-regulated status (USDA APHIS) https://www.aphis.usda.gov/aphis/ourfocus/biotechnology/permits-notifications-petitions/petitions/petition-status Am I Regulated Letters of Inquiry (USDA APHIS) https://www.aphis.usda.gov/aphis/ourfocus/biotechnology/am-i-regulated.
