# Supplementary material for: Lessons for a SECURE Future: Evaluating Diversity in Crop Biotechnology Across Regulatory Regimes
Source: Front Bioeng Biotechnol. 2022 May 2;10:886765. doi: 10.3389/fbioe.2022.886765 (PMC9108862; doi:10.3389/fbioe.2022.886765)
Supplement: Supplementary file 1 [file DataSheet1.PDF]

Methods Supplement

APPENDIX 1

This appendix provides further details and examples of how we collected data from the publicly available APHIS petitions for determination of non-regulated status (PDNS) and Am I Regulated letters of inquiry (AIR). Below readers will find more details on 1) the types of documents available in APHIS archives of regulatory submissions 2) explanation and examples of our coding scheme used for content analysis.

Document Types

Both the petitions and AIR letters of inquiry are publicly available on the USDA APHIS archive. To pull out necessary data, we downloaded all submission documents that were available in each archive and coded documents as needed to provide relevant data on developers, organisms, traits, genes, and methods. Below are the examples of the different kinds of documents available in each archive:

AIR Submissions:

- Developer Petition
- Determination of Nonregulated status
- Plant Pest Risk Assessment (PPRA)
- Environmental Impact Statement (EIS)
- Finding of No Significant Impact (FONSI)
- Environmental Assessment (EA)
- Federal Register Notice
- Record of decision
- Memorandum of Understanding (MOU)
- Memorandum of Agreement (MOA)

Note that we did not code every single document available across all submissions in both archives. Such a task would prove redundant, especially in the PDNS document archive. We usually able to garner the data we needed from looking primarily at the “developer petition” and determination of “non-regulated status” documents. These documents were fairly standardized across all submission events, which provided for a fairly efficient coding process Other documents such as plant pest risks assessments and environmental assessments provided valuable contextual information on the workings of the regulatory process, and on relevant categorical data (i.e. gene, method, trait).

In the AIR archive, there were only two main types of documents to work with; developer letters of inquiry and APHIS responses. We reviewed and coded both types to capture

all relevant categorical data. We noted that there was much overlap between the contents of both the developer's letters of inquiry and the APHIS responses, and decided that we would treat the developer submissions as our primary source of data points, and use the APHIS responses to confirm what we observed. Summaries of the proposed innovation were usually captured in the APHIS responses, allowing us to confirm data points if we couldn't find clarity on a particular data point in the developer letter.

All desired categorical data was accessible to us in the PDNS archive, but not in the AIR archive. As noted previously, there was a high degree of confidential business information (CBI) claimed in the AIR letters of inquiry. This made finding certain data points difficult, and at times impossible depending on the level of CBI claimed in a developer letter.

Below are two letters exhibiting different examples of CBI claimed. The first example (Example 1) of CBI is a letter from CoverCress Inc dated February 6th, 2020. CBI is denoted by instances of blank brackets in the text of the letter than look like "[ ]" and bolded **CBI-Deleted** in the right hand column of the letter. Both the brackets and column notations were commonly used to denote CBI across many different letters. This letter from CoverCress exhibits a common level of CBI we encountered in the letters, where both specific trait and gene target data were omitted. However, not all CBI claims omitted this much information. In example 2, a letter from the University of Minnesota dated May 15th, 2019 only claims CBI on the specific gene of interest. All other details regarding trait, method, organism, etc are disclosed (Example 2). We noted that this example from a University was not the only instance where universities claimed CBI in their letters of inquiry. For more examples of what CBI in AIR letters looks like, we encourage readers to explore the APHIS AIR archive.

Document Coding

This section of the supplement provide readers with an example of how documents were coded in our study. Across both the PDNS and AIR archive, we were coding for 5 main categories of data for each submissions; traits of interest, target genes, engineering methods, subject organism, and submitting developer. Our codebook was simple, consisting of only 5 codes corresponding to our data points of interest; trait, gene, method, organism, and developer. As we analyzed documents, we highlighted and marked text that corresponded to these data points as indicated in example 3 below.

The example below illustrates and instance of this coding process. The document is from the AIR archive, and is a developer letter submitted by Texas A&M dated September 15th, 2017. In the letter text, yellow highlights indicate relevant text that provides information on our data points of interest. We specified the code that each text excerpt corresponded to in the right hand column of the document in red.

In this letter, the developer and organism of interest were presented at the very start of the letter as "Texas A&M" and rice, respectively. Nexgen Plants Ltd is also mentioned as being in collaboration with Texas A&M, but our developer code specifically was applied to the entity

submitting the letter of inquiry. Further down the letter at the start of page two is the first mention of the “intended phenotype” which is said to be “salinity tolerance” designed to render “increased tolerance to saline growing conditions.” We marked both of these statements with the “trait” code because they pertained to the intended trait of interest for this submission. A couple paragraphs below that, under the heading “Development of Salt Tolerant Rice Cultivars” we applied both the “gene” and “method” codes. The letter indicate twice that the “rice salt tolerance gene DREB1A” as the primary target gene which was subject to “gold particle bombardment transformation.” We marked these statements with the “gene” and “method” code respectively, determining that the target gene was DREB1A and the method of engineering was biolistic transformation.

SUPPLEMENT EXAMPLE 1 (CBI)

CBI-Deleted Copy

CoverCress Inc.

RECEIVED

By apmball for BRS Document Control Officer at 2:40 pm, Feb 26, 2020

Dr. Bernadette Juarez
APHIS Deputy Administrator
Biotechnology Regulatory Services
4700 River Rd, Unit 98
Riverdale, MD 20737

February 26, 2020

Inquiry regarding APHIS position on non-segregant CRISPR/Cas9 mutant *Thlaspi arvense* L. (pennycress) lines as non-regulated articles.

Dr. Juarez,

With this letter we respectfully request confirmation from USDA-APHIS's Biotechnology Regulatory Services (BRS) that our *Thlaspi arvense* L. (pennycress; field pennycress)
CRISPR/Cas9 generated mutant lines, featuring disruption of [**CBI-Deleted**
] and [ ], are not themselves plant pests, and do not meet the **CBI-Deleted**
definition of a regulated article under 7 CFR Part 340 as the final lines do not contain any foreign or plant pest DNA. In response to our previously submitted AIR letter (dated August 15, 2019),
BRS deregulated pennycress lines harboring disruptions in [ ] **CBI-Deleted**
and [ ] genes. Mutations in [ ] and [ ] genes in these lines result in the same [**CBI-Deleted**
] and [ ] phenotypes generated by the same construct described in this **CBI-Deleted**
document. Upon confirmation of these lines as non-regulated articles we plan to field test them in multiple locations throughout Illinois and Missouri.

Mutations in the two previously mentioned genes were also introduced into pennycress cultivar
B34 using a CRISPR/SpCas9 DNA construct designed to target genomic edits to the [**CBI-Deleted**
] and [ ] genes. This triple-hit construct was delivered to B34 using a disarmed **CBI-Deleted**
Agrobacterium tumefaciens strain (GV3101) and a standard floral dip transformation method. When integrated into the plant genome, the expressed *Streptococcus pyogenes* CRISPR-associated protein 9 (SpCas9) endonuclease was guided to the three unique, targeted loci (the
[ ] and [ ] genes). At these locations, the SpCas9 endonuclease catalyzed double- **CBI-Deleted**
stranded DNA breaks, which were then repaired by the plant's error-prone endogenous NHEJ DNA repair mechanisms, resulting in heritable mutations in two of the three targeted loci.

Two of the independent plant lines that were isolated (*CC2E.1* and *CC2E.2*) were found to have mutations at the expected locations proximal to the SpCas9 NGG PAM sites adjacent to the
targeted regions in the [ ] and [ ] genes while [ ] remained unaltered. **CBI-Deleted**

The seeds of pennycress plants containing homozygous [ ] mutations have low levels of **CBI-Deleted**
[ ] which is highly accumulated in wild-type pennycress. **CBI-Deleted**
In homozygous [ ] mutants, the [ ] of pennycress are [ ] in contrast to the **CBI-Deleted**
naturally [ ] produced by wild-type pennycress, signifying the absence or reduction of **CBI-Deleted**
[ ]. These seeds contain lower levels of [**CBI-Deleted**
]. **CBI-Deleted**

CC2E.1 mutant plants harbor a single T nucleotide insertion in the [ ] gene, no **CBI-Deleted**
alteration/WT [ ] gene, and a 2-base pair deletion in the [ ] gene. **CBI-Deleted**

CC2E.2 mutant plants harbor a single T insertion in the [ ] gene, no alteration/WT [ ] **CBI-Deleted**
gene, and a 5-base pair deletion in the [ ] gene. **CBI-Deleted**

In the cases of both *CC2E.1* and *CC2E.2* triple mutant lines, the described mutations resulted in a disruption of corresponding gene functions, leading to unique and agronomically important alterations in seed composition.

The CRISPR/SpCas9 DNA construct used to deliver the mutagenesis reagent was named ‘pARV458’ and is comprised of the following 6 components:

1) The At3g11940 promoter (*RPS5A*) from *Arabidopsis thaliana* driving expression of the *Streptococcus pyogenes SpCas9* gene. The SpCas9 protein is an RNA-guided DNA endonuclease enzyme used to mutate a gene of interest. The pea3A terminator from *Pisum sativum* was used to end transcription of the *SpCas9* cassette.

2) The *U6-26* promoter from *Arabidopsis thaliana* driving expression of the *SpCas9* guide RNA sequences. The guide RNA contained 20 nucleotides of sequence identical to [ ] **CBI-Deleted** coding sequence adjacent to a NGG PAM site 288 bp downstream of the ATG start codon. This was followed by a *U6-26* terminator sequence from *Arabidopsis thaliana.*

3) The *U6-29* promoter from *Arabidopsis thaliana* driving expression of the *SpCas9* guide RNA sequences. The guide RNA contained 20 nucleotides of sequence identical to [ ] **CBI-Deleted** coding sequence adjacent to a NGG PAM site 219 bp downstream of the ATG start codon. This was followed by a *U6-26* terminator sequence from *Arabidopsis thaliana.*

4) The *U6-26* promoter from *Arabidopsis thaliana* driving expression of the *SpCas9* guide RNA sequences. The guide RNA contained 20 nucleotides of sequence identical to [ ] coding **CBI-Deleted** sequence adjacent to a NGG PAM site 455 bp downstream of the ATG start codon. This was followed by a *U6-26* terminator sequence from *Arabidopsis thaliana.*

5) The cassava vein mosaic virus (CVMV) promoter driving expression of the DsRED fluorescent protein from Discosoma (used as a fluorescent selectable marker) which was terminated with a nopaline synthase (NOS) terminator.

6) T-DNA Left Border (LB) and Right Border (RB) sequences from *Agrobacterium tumefaciens* for integration into the plant genome.

Although the original transgenic lines were generated using a construct containing plant pest sequences, the progeny were negatively selected for the presence of the transgene and the resulting null-segregant lines do not contain these non-native sequences. Therefore, we are writing to obtain confirmation that these null-segregant lines are not considered regulated articles under 7 CFR Part 340 before moving forward with planting in field test plots.

We sincerely thank you for your time and consideration.
Best,

Michaela McGinn

Michaela McGinn, PhD, and Tim Ulmasov, PhD
(636) 578-8319 (636) 891-5588
CoverCress Inc.
1100 Corporate Square Dr. Suite 135
St. Louis MO 63132

UNIVERSITY OF MINNESOTA
RECEIVED
By hdjohnson for BRS Document Control Officer at 1:38 pm, Mar 18, 2019

CBI deleted copy

Twin Cities Campus

Agronomy and Plant Genetics
College of Food, Agricultural
and Natural Resource Sciences

411 Borlaug Hall
1991 Upper Buford Circle
St. Paul, MN 55108-6026

Office: 612-625-5769
Fax: 612-625-1268
http://stuparlab.cfans.umn.edu/
Email: stup0004@umn.edu

SUPPLEMENT EXAMPLE 2 (CBI)

CBI deleted Copy

May 15, 2019

Dr. Michael J. Firko
APHIS Deputy Administrator
Biotechnology Regulatory Services
4700 River Rd, Unit 98
Riverdale, MD 20737

RE: Inquiry regarding APHIS position on non-segregant CRISPR/Cas9 mutagenized *Glycine max* (soybean) line as a non-regulated article

Dear Dr. Firko,

With this letter, we are asking the Biotechnology Regulatory Services to confirm non-regulation of null-segregant CRISPR/Cas9 mutagenized soybean lines 673-7-8 and 673-7-12 containing no transgenic sequences that was derived from a transgenic parent line 673-7. We plan to field test field test these materials at the University of Minnesota Agricultural Experiment Station in Saint Paul, Minnesota for changes in seed composition.

The transformation was carried out on Soybean (*Glycine max*) cultivar 'Bert' using a disarmed *Agrobacterium rhizogenes* strain transformed with a CRISPR associated protein 9 (Cas9) reagent described below. When integrated into the plant genome, the expressed CRISPR reagent was guided to the target locus by expressed guide RNAs to the coding region of gene models [ ]. These genes are annotated as [ ], respectively. This action induced a double-stranded break at the target sites that were repaired by the plant's DNA repair mechanism resulting in frame-shift mutations that deactivated these genes.

CBI-deleted
CBI-deleted

Null-segregant mutant lines were generated by self-pollinating the original transformed line and the screening of parent and progeny lines by PCR. We performed 20X whole genome sequencing (WGS) of the two plants 673-7-8 and 673-7-12 and found no evidence for the presence of the transgene. The T0 plant 673-7 had a transgene insertion on chromosome 1, but there was no transgene present in the progeny plants 673-7-8 and 673-7-12. The segregation of integrated transgenic material was possible since the reagent transgene was not linked to targeted loci.

The construct used to deliver the mutagenesis reagent is comprised of the following components: A *Glycine max* Ubiquitin promoter (Gmubi) driving strong constitutive expression of the *Arabidopsis thaliana* codon-optimized CRISPR associated protein 9 (Cas9). The Cas9 (CRISPR associated protein 9) is a RNA-guided DNA endonuclease enzyme used to mutate gene(s) of interest. The heatshock protein (HSP) terminator was used to end transcription of the Cas9 cassette. To drive expression of guide RNA sequences, the Cestrum yellow leaf curling virus (CmYLCV) promoter was used. To enhance

mutagenesis, a 3' repair exonuclease 2 (TREX2) driven by a figwort mosaic virus (FMV) promoter and terminated by a pea rbcsE9 terminator was also included. The guide RNAs targeted the first exon of [ ], the first exon of [ ], and the first exon of [ ]. The guide RNA cassette was terminated by the 35S terminator. The bar (bialaphos resistance) gene from *Streptomyces hygroscopicus* was used as a selectable marker for transgenic selection and was constitutively expressed by a 35S promoter from Cauliflower mosaic virus (CaMV). The bar gene encodes a phosphinothricin acetyl transferase (PAT) enzyme that detoxifies applied phosphinothricin herbicide. The 35ST_polyA from Cauliflower mosaic virus was used to terminate bar transcription. Left (LB) and Right (RB) T-DNA border sequence from *Agrobacterium tumefaciens* span the T-DNA sequence for integration into the plant genome. CBI-deleted CBI-deleted

Plant 673-7-8 maintains frameshift deletions for each of the three target genes; they are 11 bp, 5 bp, and 7 bp in length, respectively. Plant 673-7-12 maintains frameshift deletions for each of the three target genes; they are 4 bp, 5 bp, and 7 bp in length, respectively. These mutations all disrupt the complete translation of the protein in the recipient organism *Glycine max* cultivar 'Bert'. These mutants will be used to study seed composition traits. Although the original transgenic line was generated using a construct containing plant pest sequences, the resulting null segregants do not contain such sequences. Therefore, we are writing to obtain confirmation that these lines, and subsequent progeny of these lines, are not a regulated article under 7 CFR Part 340. Please confirm that this understanding is correct.

Sincerely,

Robert Stupar
Associate Professor
University of Minnesota
1991 Upper Buford Circle
411 Borlaug Hall
St Paul, MN 55018

SUPPLEMENT EXAMPLE 3 (CODING)

TEXAS A&M AGRILIFE RESEARCH

RECEIVED
By APHIS BRS Document Control Officer at 1:13 pm, Oct 13, 2017

15 September 2017

Dr. Michael J. Firko
APHIS Deputy Administrator
Biotechnology Regulatory Services
4700 River Rd, Unit 98
Riverdale, MD 20737

Re: Confirmation that salinity tolerant rice genotypes are not regulated articles

Dear Dr Firko:

Texas A&M hopes to collaborate with Nexgen Plants Pty Ltd (Nexgen), an Australian based start-up company, through field evaluations of their rice (*Oryza sativa*, L.) and by developing a number of new rice cultivars using a method that produces events that mimic natural duplication and recombination processes. Texas A&M and Nexgen respectfully seek confirmation from Biotechnology Regulatory Services that Nexgen's salinity tolerant rice does not meet the definition of a regulated article under 7 CFR Part 340. Nexgen has provided the information summarized below to assist the USDA Biotechnology Regulatory Services in making its determination. Importantly, the methodology does not introduce any foreign DNA into the plant. The entirety of the introduced DNA originates from the rice genome and no foreign DNA and no foreign marker or selection genes are introduced. Gene delivery is via particle bombardment of linearized DNA that has been gel purified.

developer
organism

Oryza sativa is not a plant pest and does not pose a weed potential. The method used to generate the salt tolerant rice results in plants with no introduced plant pest sequences, and the duplication of rice genes will not generate a plant pest or pose a weed potential. As a consequence, Nexgen considers the methodology to be a form a speed breeding and therefore the salinity tolerant rice does not meet the definition of a regulated article based on 7 CFR Part 340.

organism

The Intended Phenotype

Salinity is an important issue affecting the production of rice[1]. It affects the respiration and photosynthesis of the plants and decreases biological N_2 fixation and soil nitrogen mineralization. Other effects include:

- reduced germination rate
- reduced plant height and tillering
- poor root growth
- increased spikelet sterility
- decreased grain weight and protein content

---

[1] Dobermann A, Fairhurst T. 2000. Rice: Nutrient disorders & nutrient management. Handbook series. Potash & Phosphate Institute (PPI), Potash & Phosphate Institute of Canada (PPIC) and International Rice Research Institute. 191 p.

The intended phenotype is salinity tolerance. The introduced/duplicated rice gene(s) are associated with an increased tolerance to saline growing conditions[2,3]. **trait** **trait**

Intended Activity

Upon confirmation from APHIS-BRS that Nexgen's salinity tolerant rice cultivars are not regulated, Texas A&M intends to import the rice from Australia in accordance with USDA permits for the importation of rice and rice related articles under the authority of 7 CFR 319.55, to conduct evaluation trials for commercial traits of interest.

Developer name and contact information, including email address

Name: Peer Schek
Title: Professor
University: University of Queensland
Email: p.schenk@uq.edu.au
Phone: +61 7 3365 8817

Development of Salt Tolerant Rice Cultivars

The methodology described herein utilizes characterized rice sequences to confer salinity tolerance. The entirety of the introduced DNA originates from the rice genome. No foreign DNA and no foreign marker or selection genes are introduced.

The rice construct contains an expression cassette consisting of a rice *ACTIN* promoter and 5' untranslated region (UTR) sequence fused to a rice *DREB1A* (Dehydration-responsive element- **gene** binding protein 1A) gene coding sequence and a *DREB1A* 3' UTR and terminator sequence. This construct is a total 2375 bp in length and contains no foreign DNA sequence or single nucleotides. This rice construct was synthesized, cloned into the non-conjugative plasmid pUC57-kan[4], and sequence verified. The rice construct DNA was linearized by restriction digestion (leaving only native sequences) and separated from the plasmid backbone by agarose gel electrophoresis. The rice construct was subsequently sliced from the agarose gel, column purified, and used for rice transformation.

Nexgen generated the rice events via gold particle bombardment transformation. The resulting **method** events mimic theoretically possible naturally occurring duplication and recombination events, resulting in extra copies of the native rice salt tolerance gene *DREB1A*. The increased transcription **gene** of *DREB1A* is driven by the native rice *ACTIN* promoter, whereas termination of transcription and subsequence polyadenylation of the transcript is mediated by the native rice *DREB1A* terminator from the 3' UTR of the rice *DREB1A* gene.

---

[2] Oh, S.-J., Song, S. I., Kim, Y. S., Jang, H.-J., Kim, S. Y., Kim, M., … Kim, J.-K. (2005). Arabidopsis CBF3/DREB1A and ABF3 in Transgenic Rice Increased Tolerance to Abiotic Stress without Stunting Growth. *Plant Physiology*, *138*(1), 341–351. http://doi.org/10.1104/pp.104.059147

[3] Dubouzet, J. G., Sakuma, Y., Ito, Y., Kasuga, M., Dubouzet, E. G., Miura, S., Seki, M., Shinozaki, K. and Yamaguchi-Shinozaki, K. (2003), *OsDREB* genes in rice, *Oryza sativa* L., encode transcription activators that function in drought-, high-salt- and cold-responsive gene expression. The Plant Journal, 33: 751–763. doi:10.1046/j.1365-313X.2003.01661.x

[4] Cloning vector pUC57 kan: https://www.ncbi.nlm.nih.gov/nuccore/347984618

Rice is not a Regulated Article

Rice (*Oryza sativa*, L.), is not a federal noxious weed pursuant to 7 CFR 360. **organism**

No Plant Pest Risk or Increased Weed Potential

Although weedy red rice can be a problematic weed in cultivated rice[5,6], we do not believe that the salinity tolerant rice will have a significant environmental impact. Firstly, both cultivated rice and red rice are predominantly self-pollinating; however, some cross pollination (<1%) does occur[7,8,9]. Thus, genes for enhanced traits in rice could potentially transfer to weedy relatives, including red rice. However, control practices are available to mitigate introgression of salinity tolerance into red rice should it occur. **trait** As such there would be no impact relating to outcrossing of the salinity tolerant rice. Secondly, *Oryza sativa* does not persist in unmanaged ecosystems as it can only survive where rice is cultivated and can be managed with existing agricultural practices. Lastly, there is unlikely to be any effects to non-target organisms including beneficial, threatened or endangered species because the salinity tolerance is conferred by a rice gene that is not known to have toxic properties. **trait** Further, the introduced DNA does not lead to the production of any novel protein that may be toxic or allergenic.

Conclusions

Nexgen has developed a methodology to generate salinity tolerant rice using DNA sequences only **trait** from the rice genome. The salinity tolerant rice contains no plant pest sequences, and the **trait** phenotype is highly unlikely to result in increased weediness or plant pest potential.

Prior to further investigation of both the method and product development of salinity tolerant rice, Texas A&M and Nexgen requests confirmation from Biotechnology Regulatory Services that the salinity tolerant rice (*Oryza sativa*) does not meet the definition of a regulated article under 7 CFR **organism** Part 340.

Sincerely,

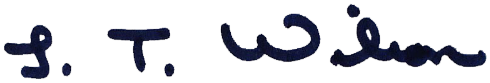

Professor, Center Director, and Endowed Chair in Rice Research

Texas A&M AgriLife Research

---

[5] Vinod K. Shivrain, Nilda R. Burgos, Robert C. Scott, Edward E. Gbur, Leopoldo E. Estorninos, Marilyn R. McClelland, Diversity of weedy red rice (*Oryza sativa* L.) in Arkansas, U.S.A. in relation to weed management, Crop Protection, Volume 29, Issue 7, 2010, Pages 721-730, ISSN 0261-2194, http://dx.doi.org/10.1016/j.cropro.2010.02.010.

[6] J.C. Delouche, N.R. Burgos, D.R. Gealy, M.G. Zorilla-San, R. Labrada, M. Larinde Weedy Rices: Origin, Biology, Ecology and Control FAO of the United Nations, Rome (2007) 144 pp

[7] D.R. Gealy, D.H. Mitten, J.N. Rutger Gene flow between red rice (*Oryza sativa*) and herbicide-resistant rice (O. sativa): implications for weed management Weed Technol., 17 (2003), pp. 627-645

[8] Q. Cao, B.R. Lu, H. Xia, J. Rong, F. Sala, A. Spada, F. Grassi Genetic diversity and origin of weedy rice (*Oryza sativa f. spontanea*) populations found in north-eastern China revealed by simple sequence repeat (SSR) markers Ann. Bot., 98 (2006), pp. 1241-1252

[9] V.K. Shivrain, N.R. Burgos, M.M. Anders, S.N. Rajguru, J.W. Moore, M.A. Sales Gene flow between Clearfield™ rice and red rice Crop Prot., 26 (2007), pp. 349-356

APPENDIX 2

SECURE exempt/regulated category	Regulated	Database values
contains a single non-template-guided gene edit	N	"GENES IN LINE" = 1 or 0
		AND "EDITING" != N
		AND "CRISPR REPAIR" = N
null segregant of a engineered line with no remaining engineered genes	N	"NULL SEGREGANT" = Y
		AND "GENES IN LINE" = 1 or 0
Insertion of a single gene from the same species as host organism	N	"GENES IN LINE" = 1 or 0
		AND donor species recorded for gene of interest is the same as target
Repetition of prior Plant-Trait-Mode of Action	N	SPECIES is not the first occurrence AND is not the first occurrence of any USDA P-T-MOA

TABLE 1 – Exemption Categories

An important part of this study was to use the past regulatory submission data to forecast how the biotech regulatory landscape under the new SECURE rule would treat different product types. To do this, we analyzed how many of the 303 regulatory submissions from AIR and PDNS would have qualified for an exemption category or proceeded to RSR. For analysis of projected outcomes under the SECURE rule, we characterized each entry in our database as a predicted exemption when corresponding to a set of values, as specified in Table 1 (see Methods Supplement). Outputs indicated which events in AIR and PDNS submissions would be "regulated" and required an RSR or "exempted" based on a number of categorizations for novel products. Categorizations (column 1) that would indicate an exempt product include: 1) if a product contains a single, non-template-gudied gene edit, 2) a product is a null segregant of an engineered line with no remaining engineered genes, 3) a product contains an insertion of a single gene from the same species as the host organism or 4) a product is a repetition of a previously exempt plant-trait-mode of action combination. We entered these categories into our database used to generate figure 8 according to the values in column 3.
